# Going on up to the SPIRIT in AI: will new reporting guidelines for clinical trials of AI interventions improve their rigour?

**DOI:** 10.1186/s12916-020-01754-z

**Published:** 2020-09-09

**Authors:** Paul Wicks, Xiaoxuan Liu, Alastair K. Denniston

**Affiliations:** 1 Wicks Digital Health, Lichfield, Staffordshire UK; 2 Institute of Inflammation and Ageing, College of Medical and Dental Sciences, University of Birmingham, Birmingham, UK; 3 University Hospitals Birmingham NHS Foundation Trust, Birmingham, UK; 4 Health Data Research UK, London, UK; 5 Moorfields Eye Hospital NHS Foundation Trust, London, UK; 6 National Institute of Health Research BRC for Ophthalmology, Moorfields Eye Hospital NHS Foundation Trust, London, UK; 7 Centre for Regulatory Science and Innovation, Birmingham Health Partners, Birmingham, UK

**Keywords:** Clinical trial, Machine learning, Artificial intelligence, Reporting guidelines, Checklist

## Background

In September of 2019, British Prime Minister Boris Johnson posed a dystopian conundrum to the United Nations General Assembly: “AI, what will it mean? Helpful robots washing and caring for an aging population, or pink-eyed terminators sent back from the future to cull the human race?” [1]. Amongst the hyperbole, Johnson posed a question that medicine must address: “Can these algorithms be trusted with our lives and hopes? Should the machines—and only the machines—decide... what surgery or medicines we should receive?... And how do we know that the machines have not been insidiously programmed to fool us or even to cheat us?”

### Flattening the hype curve in AI

While it has been recognized that AI may have been “overhyped” [2], today AI algorithms are increasingly involved in drug discovery, symptomatic triage, breast cancer screening, predicting acute kidney injury, and even offering mental health support. However, a recent systematic review of over 20,000 medical imaging AI studies found concerning issues of bias, lack of transparency, or inappropriate comparator groups, which meant that < 1% of those studies were of sufficient quality to be considered a trustworthy evaluation of the algorithm [3]. A year after Johnson’s provocation, a global multidisciplinary coalition has convened to address these shortcomings and take us towards the “plateau of productivity” [2] of the hype cycle for AI by setting new standards that encourage researchers, journals, and funders to open up the black box and establish public trust.

Over the course of 18 months, the consortium rigorously developed extensions to two of the most trusted minimum reporting guidelines in medicine: Standard Protocol Items: Recommendations for Interventional Trials (SPIRIT) and Consolidated Standards of Reporting Trials (CONSORT). In brief, SPIRIT is the international standard for reporting of protocols of randomized clinical trials—i.e. what you intended to do—and CONSORT is the international standard for reporting of the delivery and results of those trials—i.e. what you actually did.

These new recommendations involved a process for systematically gaining consensus from 169 international stakeholders, identifying areas of particular importance involving AI interventions that are not currently covered by the existing guidelines. The SPIRIT-AI and CONSORT-AI checklists contain 15 and 14 new items respectively as extensions to the existing SPIRIT 2013 and CONSORT 2010 checklists. The guidelines include requirements for reporting of areas such as the quality and completeness of input data, and investigation of error cases, defining the clinical context and the human-AI interaction involved.

On September 9, 2020, the SPIRIT-AI and CONSORT-AI extensions were published simultaneously in Nature Medicine, the BMJ, and Lancet Digital Health [4, 5], with authors including regulators (FDA and MHRA) and editors of many of the leading medical journals. The hope is that the guidelines will better position journal editors, peer reviewers, and journal readers, who might be expert in clinical research or medical practice but less informed about AI to know what questions to ask of a manuscript in this field, to spot what is missing (whether intentional or not), to be better equipped to evaluate the quality of a study, and to make decisions based on its results. By demonstrating ‘what good looks like’ it is hoped that these standards will lead to improved standards of design, delivery and reporting of trials in this area, and increase the impact of high quality studies through their greater visibility.

Strengths of the approach include the involvement of patient partners, a systematic Delphi process, and broad participation from across the medical technology industry, academia, and big tech firms. Important recommendations with broad applicability include the specification of “intended use” for particular algorithms (CONSORT-AI 1b), which helps give specificity to the aspect of a study that involves AI, such as highlighting medical images. Transparently defining the biases of input data sources (CONSORT-AI 4a ii) and how missing data are to be addressed (CONSORT-AI 5 iii) will avoid accusations of cherry-picking which could lead to less generalizable findings.

Challenges remain, however. These guidelines will only have value if they are followed, and we know that adherence to existing CONSORT guidelines have been variable in practice, with an audit study of leading high-impact journals finding “extensive misunderstandings” about correct outcome reporting [6]. Many AI studies are presented not in clinical medicine journals but as non-peer-reviewed conference proceedings at computer science conferences, or may enter the public domain through preprint servers such as MedRxiv. While the use of version numbers is useful in establishing which exact iteration of a codebase was deployed for an algorithm, further development of an algorithm in the future might have unpredictably different performance, and progressively self-improving algorithms could go awry in their performance outside of controlled settings. Consumer technologies such as social networks, smartphone apps, and smart devices may all use AI approaches developed outside the context of a randomized controlled trial yet have a significant impact on patients by targeting them with direct-to-consumer advertising, or monitoring their well-being in an unregulated context—the existence of these guidelines cannot offer blanket reassurance to the public that all medical AI is operating safely or transparently. Finally it is yet to be seen how commercial organisations that rely upon proprietary training data sets or carefully iterated algorithms will be able to adhere to academic standards of transparency while maintaining their fiduciary responsibilities to investors, employees, and partners.

Future work is already underway to improve the standard of design and reporting for non-randomized studies including retrospective observational analysis and the development of prognostic models that depend upon AI. This work will soon lead to EQUATOR-supported guidelines specifically for both diagnostic test accuracy studies (STARD-AI [7]) and prognostic model evaluations (TRIPOD-ML [8]).

## Conclusions

While it remains early days, these are positive signs for a maturing field. We hope to systematically advance the interactions between humans and AI in medicine by increasing the number of people who can reliably “trust but verify” the work of this rapidly expanding field.

## Data Availability

N/A.
